# Tumor-associated neutrophils and survival outcomes in colorectal cancer: a systematic review and multilevel meta-analysis

**DOI:** 10.3389/fonc.2026.1788605

**Published:** 2026-02-25

**Authors:** Ran Yang, Chongwei Zhu, Jinjin Zhao, Yun Tian

**Affiliations:** 1 Department of Oncology, Jiangsu Province Hospital of Chinese Medicine, Affiliated Hospital of Nanjing University of Chinese Medicine, Nanjing, Jiangsu, China; 2 The First Clinical Medical College, Nanjing University of Chinese Medicine, Nanjing, Jiangsu, China

**Keywords:** cancer-specific survival, colorectal cancer, disease-free survival, meta-analysis, overall survival, tumor microenvironment, tumor-associated neutrophils

## Abstract

**Objective:**

Tumor-associated neutrophils (TANs) are abundant in the colorectal cancer (CRC) microenvironment, but their prognostic relevance remains controversial. We conducted an updated systematic review and multilevel meta-analysis to evaluate associations between TAN infiltration in primary tumors and survival outcomes in CRC.

**Methods:**

PubMed, Embase, Web of Science, and the Cochrane Library were searched from inception to January 1, 2026. We included studies of histologically confirmed CRC that quantified TANs in primary tumors and reported hazard ratios (HRs) for cancer-specific survival (CSS), overall survival (OS), or disease-free survival (DFS); recurrence-free survival was combined with DFS. Multilevel random-effects models accounted for correlated effect sizes and generated overall and region-specific estimates from univariate and multivariate analyses. Prespecified subgroup analyses and multilevel meta-regression explored heterogeneity by study region, tumor stage, detection method, and TAN markers.

**Results:**

Twenty-five studies (10,356 patients) were included. High TAN infiltration was consistently associated with improved CSS (univariate HR 0.58, 95% CI 0.48–0.71; multivariate HR 0.66, 95% CI 0.54–0.81) with low heterogeneity across tumor regions. For OS and DFS, pooled estimates suggested an overall protective trend with substantial heterogeneity; subgroup analyses indicated stronger protective associations in Euro–American cohorts and in stage I–III disease. In contrast, multivariate DFS models suggested higher recurrence risk in Euro–American populations and in studies with unclear TAN marker reporting. Meta-regression identified tumor stage and TAN markers as major contributors to heterogeneity in OS and DFS.

**Conclusions:**

High TAN infiltration is a robust, independent favorable factor for cancer-specific survival in CRC. Associations with OS and DFS are context-dependent and appear modified by tumor stage and methodological factors, particularly TAN markers and detection methods, supporting refined prognostic stratification and further study of neutrophil-targeted strategies.

**Systematic Review Registration:**

https://www.crd.york.ac.uk/prospero/display_record.php?ID=CRD42023493604, identifier CRD42023493604.

## 1 Introduction

According to the latest global cancer statistics released in April 2024, colorectal cancer (CRC) accounts for approximately 9.6% of all new cancer cases and 9.3% of cancer-related deaths worldwide (1). The global burden of CRC is substantial and continues to rise, particularly in low- and middle-income countries (LMICs) (2). Early identification of reliable prognostic biomarkers and timely interventions are crucial for improving survival and quality of life for patients with CRC (3, 4).

Neutrophils, the most abundant circulating leukocytes in humans, constitute the first line of immune defense against infection and inflammation (5). Since the earliest report in 1970 suggesting a negative correlation between peripheral blood neutrophil counts and cancer prognosis (6), these cells have attracted growing attention in oncology. Neutrophils that infiltrate tumor tissues, known as tumor-associated neutrophils (TANs) (7), are involved in key processes such as the cancer–immune cycle, immune editing, and immune evasion (8). Despite their important role in the tumor immune landscape, the prognostic relevance of TANs remains uncertain. Studies assessing TANs as independent prognostic factors in solid tumors have yielded conflicting results: high TAN infiltration has been linked to poor outcomes in hepatocellular carcinoma (9), renal cell carcinoma (10), and melanoma (11), but shows no significant association in gastric (12) or head-and-neck squamous cell carcinoma (13). Conversely, in non–small-cell lung cancer, greater TAN infiltration has been associated with prolonged survival (14, 15). Notably, even within the same tumor type, the prognostic impact of TANs remains inconsistent, particularly in CRC (16–18).

To clarify the inconsistent findings regarding the prognostic role of TANs in CRC, we conducted a multilevel meta-analysis. A hierarchical random-effects model with robust variance estimation was used to integrate correlated effect sizes across studies, yielding more reliable pooled estimates. This analysis elucidates the prognostic significance of TAN infiltration in CRC and provides a quantitative basis for refining risk stratification and guiding future clinical and immunological investigations.

## 2 Materials and methods

### 2.1 Protocol and registration

This meta-analysis was designed and reported in accordance with the Preferred Reporting Items for Systematic Reviews and Meta-Analyses (PRISMA 2020) guidelines (19). A detailed PRISMA checklist is provided in **Supplementary Table 1**. The study protocol was prospectively registered in the International Prospective Register of Systematic Reviews (PROSPERO; registration ID CRD42023493604).

### 2.2 Search strategy

A comprehensive literature search was performed in PubMed, Embase, Web of Science, and the Cochrane Library to identify studies published from database inception through January 1, 2026. The search strategy was developed according to the Population–Intervention–Comparator–Outcome (PICO) framework, as summarized in **Supplementary Table 2**. Detailed search strategies for each database are presented in **Supplementary Table 3**. All retrieved records were screened in two stages: (i) title and abstract review and (ii) full-text assessment against the predefined inclusion and exclusion criteria.

### 2.3 Inclusion and exclusion criteria

Studies were eligible for inclusion if they met all of the following criteria: (i) enrolled patients with histopathologically confirmed CRC; (ii) assessed TAN infiltration in primary tumor tissue using histopathological evaluation or transcriptomic estimation; (iii) reported the association between TAN levels and at least one survival outcome, including cancer-specific survival (CSS), overall survival (OS), or disease-free survival (DFS); studies reporting recurrence-free survival (RFS) were also included and analytically grouped with DFS; (iv) provided sufficient information to extract or calculate a hazard ratio (HR) with its 95% confidence interval (CI); and (v) were published as full-text articles in English. Exclusion criteria were as follows: (i) non-original publications, including reviews, editorials, letters, conference abstracts, and case reports; (ii) non-human studies; (iii) studies with overlapping or duplicate patient populations (with the largest or most complete dataset retained); (iv) studies with a sample size < 50 patients; or (v) studies lacking adequate data for effect size estimation.

### 2.4 Data extraction

A standardized data extraction form was used to collect key information from each eligible study. The extracted variables included: first author; year of publication; country; study period; sample size (and the number of patients evaluated for TANs); follow-up duration; tumor stage reported using either the Union for International Cancer Control (UICC) TNM classification or the Dukes staging system (with Dukes A–D broadly corresponding to UICC stages I–IV); receipt of preoperative treatment; TAN assessment method; TAN-related markers; cut-off criteria or methods for defining high versus low TAN density; cut-off values; TAN localization; survival outcomes (CSS, OS, and DFS); and data source (univariate and/or multivariate analysis). Studies reporting RFS were grouped with DFS as a unified time-to-event outcome based on overlapping endpoint definitions (**Supplementary Table 4**).

TAN localization was classified into four predefined groups. Whole-tumor section (WTS) referred to a global, section-level assessment of TAN density across the entire tumor tissue without regional subdivision into tumor nest (TN), tumor stroma (TS), or invasive margin (IM); TN was defined as intraepithelial tumor-infiltrating neutrophils within tumor nests; TS represented the stromal compartment surrounding tumor nests; and IM referred to the tumor–host interface at the invasive margin. TAN density was dichotomized into high and low levels according to the definitions used in the original studies. For meta-analytic modeling, “overall” effects were obtained by jointly modeling all available region-specific effect sizes (WTS, TN, TS, and IM), whereas region-specific analyses were conducted separately for each anatomical category.

For studies in which survival outcomes were reported only as Kaplan–Meier curves, survival probabilities at multiple time points were digitized using Engauge Digitizer (version 4.1) to enable reconstruction of time-to-event estimates. Hazard ratios (HRs) and corresponding 95% confidence intervals were then reconstructed according to the methods described by Tierney et al. (20), based on the estimated numbers at risk, events, and log-rank statistics where available. When essential information was incomplete, attempts were made to contact the corresponding authors; studies were excluded if no response or usable data were obtained. Data extraction was independently performed by two authors (R. Yang and C. Zhu), with discrepancies resolved by discussion with additional researchers.

### 2.5 Quality assessment

The methodological quality of the included studies was evaluated using the Newcastle–Ottawa Scale (NOS) (21). This scale comprises three domains: selection (0–4 points), comparability (0–2 points), and outcome assessment (0–3 points). Two reviewers independently assessed study quality, and disagreements were resolved by discussion. Studies with NOS scores of ≥7 were considered high quality.

### 2.6 Statistical analysis

All statistical analyses were performed in R (version 4.5.0). For each study, HRs and corresponding 95% CIs were extracted or derived. HRs were inverted when necessary to ensure that HR < 1 uniformly indicated a favorable prognosis associated with higher TAN infiltration. The main survival outcomes were CSS, OS, and DFS; studies reporting RFS were analytically grouped with DFS. Statistical significance was defined as a 95% CI not crossing 1.

Univariate and multivariate HRs were synthesized separately. Because many studies reported multiple correlated effect sizes from different tumor regions (WTS, TN, TS, IM) or from multiple cohorts within the same publication, a multilevel random-effects model (rma.mv, REML) was applied, with effect sizes nested within studies (Study_ID/Effect_ID). Between-study and within-study variance components were estimated simultaneously, and heterogeneity was quantified using an I²-like statistic. For the main analysis, all available effect sizes from WTS, TN, TS, and IM were combined to obtain an overall pooled estimate, and region-specific analyses were conducted separately for each anatomical category. Pooled HRs were reported with 95% CIs and 95% prediction intervals (PIs).

When substantial heterogeneity was present (I²-like > 50%), prespecified subgroup analyses and meta-regression were performed to explore potential moderators. Moderators included study region, sample size, tumor stage, preoperative treatment status, TAN detection method, and TAN marker type. Meta-regression was conducted only when ≥10 studies were available, using multilevel random-effects models with cluster-robust variance estimators and CR2 small-sample correction to obtain robust standard errors (robust SEs). A P value < 0.05 for subgroup comparisons or regression coefficients was considered statistically significant.

Sensitivity analyses included leave-one-out procedures and influence diagnostics. Robustness was further evaluated by applying CR2-based robust SEs and by repeating analyses after excluding studies with lower quality, smaller sample sizes, or differing TAN assessment methods or markers. Publication bias was assessed using funnel plots, Egger’s regression, and Begg’s rank correlation tests; these tests and the trim-and-fill method were applied when ≥10 studies were available for a given outcome. All tests were two-sided, with P < 0.05 considered statistically significant.

## 3 Results

### 3.1 Search results

**Figure 1:**
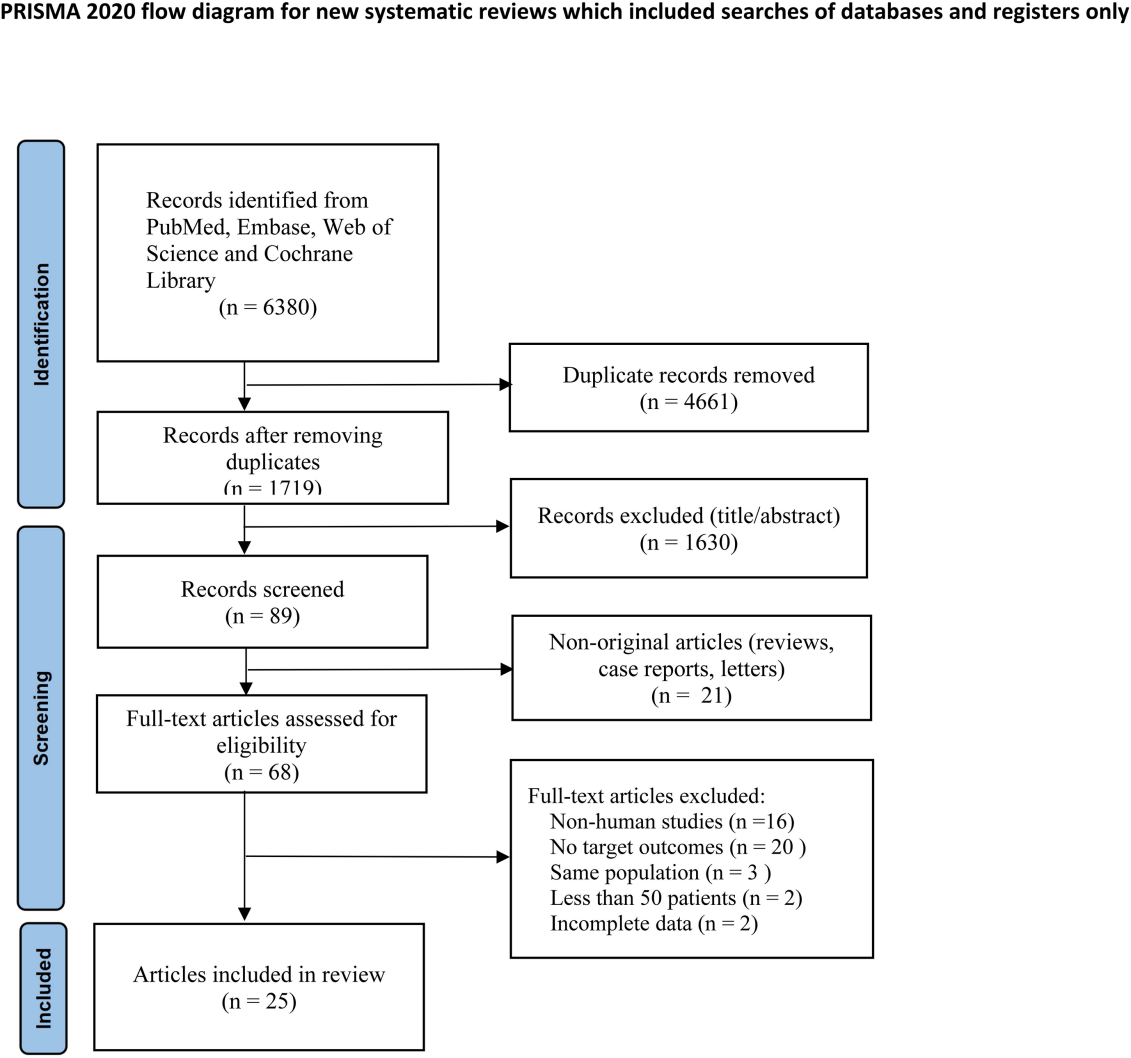
PRISMA flow diagram of study selection.

A total of 6,380 records were identified through the database search. After removing 4,661 duplicates and screening titles and abstracts, 89 records remained, of which 21 were excluded as reviews, case reports, or letters. The remaining 68 full-text articles were assessed against the predefined eligibility criteria, and 43 were excluded. Ultimately, 25 studies fulfilled the inclusion criteria and were included in the meta-analysis (**Figure 1**).

### 3.2 Study characteristics and quality assessment

This meta-analysis included 25 studies involving 10,356 patients with CRC, published between 2005 and 2025 (22–46). Geographically, 12 studies were conducted in Euro–American populations, 12 in East Asia (China and Japan), and one study analyzed publicly available transcriptomic datasets. Patient cohorts covered tumor stages I–IV, reported using either the UICC TNM classification or the Dukes staging system, with Dukes A–D broadly corresponding to UICC stages I–IV. Regarding preoperative treatment, 11 studies exclusively included treatment-naïve patients, 3 included patients who had received preoperative radiotherapy or chemotherapy, and 11 did not report preoperative treatment status. Detailed clinical and methodological characteristics of the included studies are summarized in **Table 1**.

**Table 1:** Clinical and methodological characteristics of the included studies.

Author (year)	Country	Study period	Sample size (n)	Follow-up (median, months)	Male	Age (median, years)	Tumor stage	Preoperative treatment	TAN detection method	TAN marker	Cut-off criterion or method	Cut-off value	TAN location	Survival outcomes	Analysis type	NOS
Xia (2025) (22)	China	2015	110	NR (range 1–64)	70	62	I-III	NR	IHC	CD66b	Median	15cells/HPF	WTS	OS	Univariate	8
Rottmann (2023) (23)	USA	2011-2017	348	NR	164	65 (mean)	I-IV	NR	H&E	NA	Morphologic pattern (NR/NI/NP)	NR vs NI/NP	TN	RFS	Uni- and multivariate	8
Jakubowska (2022) (24)	Poland	2014-2016	160	60	96	68 (mean)	I-IV	Yes	H&E	NA	TN: presence/absence; TS: proportion threshold	TN presence; TS 21% threshold	TN, TS	DFS	Univariate	7
Yin (2022) (25)	Japan	2013-2015	103	NR	63	71	I-III	No	IHC	CD66b	ROC (Youden index)	77 cells/HPF	IM	DFS, OS	Univariate	7
Zhang (2022) (26)	China	NR	93	NR	52	68	I-IV	NR	IHC	MPO	Proportion of MPO positive cells	MPO score: 1–3 vs. 0	TS	OS	Uni- and multivariate	9
Xu (2021) (27)	China	2009-2012	1021	58	623	61 (mean)	I-III	NR	IHC	CD66b	StepMiner algorithm	60 cells	WTS	DFS, OS	Uni- and multivariate	8
Mehrabi (2021) (28)	Sweden	1990	62	NR	33	NR	I–III	No	IHC	CD66b	ROC (Youden index)	8 cells per TMA core	WTS	OS	Univariate	9
Vayrynen (2020) (29)	USA	1976-2014	934	NR	413	NR	I-IV	NR	H&E	NA	G-cross function (upper quartile)	Upper quartile (75th percentile)	TN, TS	CSS, OS	Uni- and multivariate	9
Ye (2019) (30)	China	2001-2011	1008 (training/testing/validation: 359/249/400)	NR	596 (training/testing/ validation: 205/165/226)	NR	I-III	NR	IHC	CD66b	Median	NR	WTS	DFS, OS	Uni- and multivariate	8
Edin (2019) (31)	Finland	1998-2003	275	NR	143	69	I-IV	Yes	IHC	CD66b	Median	TN 31.7; TS 95.8 cells/mm²	TN, TS	CSS	Uni- and multivariate	8
Hu (2019) (32)	China	2007-2009	276	NR	164	57 (mean)	I-IV	NR	IHC	CD66b (CEACAM8)	X-tile program	17 cells per TMA core	WTS	DFS, OS	Univariate	8
Zhu (2018) (33)	China	2006-2007 (training); 2008-2009 (validation)	582 (training/ validation: 337/245)	NR	353 (training/ validation: 208/145)	NR	I-IV	No	IHC	CD66b	Median	NR	WTS	DFS, OS	Uni- and multivariate	7
Xiong (2018) (34)	NA	Up to 2017	1011	NR	NA	NA	I-IV	NR	Transcriptomic deconvolution	NA	Median	NR	WTS	DFS	Uni- and multivariate	7
Zhou (2018) (35)	China	2008-2010	378	66	NA	NR	I-IV	No	IHC	CD177	Median	8 cells/HPF	WTS	DFS, OS	Uni- and multivariate	8
Governa (2017) (36)	Switzerland	NR	677	NR	321	69 (mean)	I-III	NR	IHC	CD66b	Regression tree analysis	10 cells per punch	TN	OS	Univariate	7
Berry (2017) (37)	USA	1991-2014	221	68	107	61 (mean)	I-IV	No	Manual count	NA	Median	5.4 cells per 20 fields	TN	OS	Univariate	7
Wikberg (2017) (38)	Sweden	1995-2003	448	NR	TN: 224; IM: 213	NR	I-IV	Yes	IHC	CD66b	Semi-quantitative scored	High 2-4	TN, IM	CSS	Univariate	8
Galdiero (2016) (39)	Italy	1997-2006	128	58 (mean)	73	NR	I-IV	No	IHC	CD66b	Immunoreactive area (median values)	TN: 0.17; IM: 0.11	TN, IM	CSS, DFS	Univariate	8
Chen (2016) (40)	China	2000-2006	300	63(mean)	NR	NR	I-IV	No	IHC	CD15	X-tile program	NR	WTS	DFS, OS	Univariate	8
Lin (2015) (41)	China	2008	78	NR	46	NR	Dukes A–D	No	IHC	CD15	50th percentile	31 cells/HPF	TN	OS	Univariate	7
Droeser (2013) (42)	Switzerland	NR	1192 (training/ validation: 609/583)	68	1016 (training/ validation: 501/515)	71	I-IV	No	IHC	MPO	Regression tree analysis	60 cells per punch	TN	OS	Univariate	8
Rao (2012) (43)	China	2000-2006	229	60	142	57.3	I-IV	No	IHC	CD66b	ROC curve analysis	60 cells per TMA spot	TN	OS	Uni- and multivariate	8
Richards (2012) (44)	UK	1997-2006	130	105	68	NR	I-III	No	H&E	NA	Median	15 cells per 0.018 mm^2^	IM	CSS	Univariate	7
Khanh (2011) (45)	Japan	1998-2005	206	NR	114	63(mean)	I-III	NR	IHC	CD10	Semi-quantitative score (0-3)	High: score 2-3; low: score 0-1	IM	RFS, OS	Univariate	7
Klintrup (2005) (46)	Finland	1986-1996	386	41 (mean)	179	67 (mean)	Dukes A–D	NR	H&E	NA	Two-tier dichotomization	High: grade 2-3; low: 0-1	TN	OS	Univariate	7

WTS refers to a whole-tumor section (entire tumor tissue without subdivision into TN, TS, or IM). Tumor stage was reported using either the UICC TNM classification or the Dukes staging system; Dukes A–D broadly correspond to UICC stages I–IV. Recurrence-free survival (RFS) was considered conceptually comparable to disease-free survival (DFS) and was combined with DFS in the meta-analysis. In Rottmann et al., NR, NI, and NP denote neutrophil-rich (>15 TANs per 100 tumor cells), neutrophil-intermediate (5–15), and neutrophil-poor (<5), respectively.

TAN, tumor-associated neutrophils; NR, not reported; NA, not available; TN, tumor nest; TS, tumor stroma; IM, invasive margin; WTS, whole-tumor section; TMA, tissue microarray; HPF, high-power field; IHC, immunohistochemistry; H&E, hematoxylin and eosin staining; ROC, receiver operating characteristic curve; CSS, cancer-specific survival; OS, overall survival; DFS, disease-free survival; RFS, recurrence-free survival; CD66b, neutrophil surface antigen also known as CEACAM8; MPO, myeloperoxidase; CD15, Lewis X antigen; CD10, neutral endopeptidase; CD177, neutrophil-specific glycoprotein; UICC, Union for International Cancer Control; NOS, Newcastle–Ottawa Scale.

A range of methodological approaches was used to quantify TANs. Immunohistochemistry (IHC) was the predominant technique (18 studies), employing markers such as CD66b (CEACAM8), MPO, CD15, CD177, and CD10. Five studies used hematoxylin–eosin (H&E)–based morphological assessment, one used manual cell counting, and one utilized transcriptomic deconvolution. Cut-off definitions for high versus low TAN density varied across studies and were based on median values, ROC curve analysis (Youden index), X-tile software, regression tree analysis, semi-quantitative scoring, or predefined absolute cell-count thresholds.

TAN localization was assessed in four predefined anatomical compartments: WTS, TN, TS, and IM. Nine studies evaluated WTS, 12 focused on TN, 4 on TS, and 5 on IM. Survival outcomes included CSS, OS, and DFS; studies reporting RFS were analytically grouped with DFS.

All included studies demonstrated high methodological quality, with NOS scores ranging from 7 to 9, indicating an overall low risk of bias.

### 3.3 Cancer-specific survival

#### 3.3.1 Overall analysis (all regions combined)

**Figure 2:**
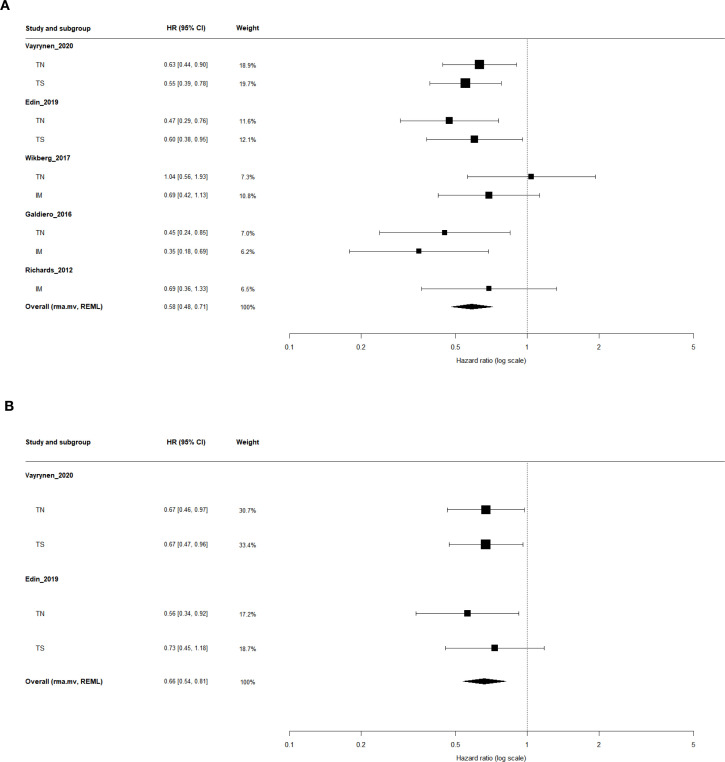
Forest plots of the association between tumor-associated neutrophil (TAN) infiltration and cancer-specific survival (CSS). **(A)** Univariate analysis. **(B)** Multivariable analysis adjusted for clinicopathological covariates. Region-specific effect sizes (tumor nest [TN], tumor stroma [TS], and invasive margin [IM]) were pooled using a multilevel random-effects model (rma.mv, REML) to account for the within-study correlation of multiple tumor regions. Hazard ratios (HRs; log scale) < 1 indicate improved survival in patients with high versus low TAN infiltration.

In the univariate analysis, five independent studies (nine effect sizes; n = 1,915) evaluated the association between TAN infiltration and CSS. The pooled estimate obtained from a multilevel random-effects model (rma.mv, REML) showed that high TAN infiltration was significantly associated with improved CSS (HR = 0.58; 95% CI, 0.48–0.71; 95% PI, 0.43–0.79; I²-like = 15.7%; **Table 2A**, **Figure 2A**), accompanied by low between-study heterogeneity.

**Table 2:** Pooled hazard ratios for TAN infiltration in overall and tumor localization–specific analyses.

(A) Univariate models											
Survival outcome	Tumor region	No. of effects	No. of studies	Pooled HR (95% CI)	P-value	Prediction interval	τ² (between)	τ² (within)	I²-like (total)	I²-like (between)	I²-like (within)
CSS	Overall	9	5	0.58 (0.48-0.71)	<0.001	0.43-0.79	0.014	0	15.7	15.7	0
	TN	4	4	0.60 (0.44-0.81)	0.001	0.38-0.95	0.016	0.015	29.5	14.8	14.8
	TS	2	2	0.57 (0.43-0.75)	<0.001	0.43-0.75	0	0	0	0	0
	IM	3	3	0.57 (0.38-0.86)	0.007	0.33-1.00	0.019	0.019	28.1	14.1	14.1
OS	Overall	23	18	0.79 (0.53-1.16)	0.222	0.17-3.73	0.592	0	81.1	81.1	0
	WTS	11	8	0.70 (0.37-1.33)	0.279	0.11-4.39	0.769	0	84.1	84.1	0
	TN	8	7	0.93 (0.56-1.56)	0.785	0.24-3.62	0.411	0	81.8	81.8	0
	TS	2	2	0.78 (0.42-1.42)	0.415	0.31-1.92	0.059	0.059	50.5	25.2	25.2
	IM	2	2	0.42 (0.03-5.19)	0.502	0.01-28.36	1.483	1.483	90.8	45.4	45.4
DFS	Overall	17	12	0.73 (0.42-1.28)	0.272	0.10-5.14	0.909	0	91.1	91.1	0
	WTS	10	7	0.60 (0.27-1.32)	0.202	0.07-5.28	1.071	0	92.7	92.7	0
	TN	3	3	1.20 (0.37-3.84)	0.760	0.12-11.52	0.490	0.490	92.7	46.3	46.3
	TS	1	1	–	–	–	–	–	–	–	–
	IM	3	3	0.59 (0.30-1.16)	0.125	0.18-1.92	0.121	0.121	66.4	33.2	33.2

(B) Multivariate models										
Survival outcome	Tumor region	No. of effects	Pooled HR (95% CI)	P-value	Prediction interval	τ² (between)	τ² (within)	I²-like (total)	I²-like (between)	I²-like (within)
CSS	Overall	4	0.66 (0.54-0.81)	<0.001	0.54-0.81	0	0	0	0	0
	TN	2	0.63 (0.47-0.85)	0.002	0.47-0.85	0	0	0	0	0
	TS	2	0.69 (0.52-0.92)	0.011	0.52-0.92	0	0	0	0	0
	IM	0	–	–	–	–	–	–	–	–
OS	Overall	10	0.80 (0.38-1.71)	0.569	0.10-6.17	0.933	0	85.4	85.4	0
	WTS	6	0.50 (0.16-1.51)	0.217	0.05-5.30	1.137	0	83.4	83.4	0
	TN	2	1.14 (0.38-3.39)	0.816	0.18-7.17	0.287	0.287	92.4	46.2	46.2
	TS	2	1.20 (0.36-4.01)	0.772	0.16-9.16	0.349	0.349	91.2	45.6	45.6
	IM	0	–	–	–		–	–		
DFS	Overall	8	0.67 (0.27-1.62)	0.370	0.07-6.70	1.053	0.129	92.1	82.0	10.1
	WTS	7	0.54 (0.21-1.41)	0.209	0.05-5.51	1.022	0.143	91.7	80.4	11.2
	TN	1	–	–	–	–	–	–	–	–
	TS	0	–	–	–	–	–	–	–	–
	IM	0	–	–	–	–	–	–	–	–

All pooled estimates were obtained using multilevel random-effects models (rma.mv, REML), with effect sizes nested within studies when multiple cohorts or tumor regions were reported. “Overall” denotes models jointly including all available tumor localizations (WTS, TN, TS, IM). Tumor localization–specific rows correspond to individual compartments. DFS includes studies reporting recurrence-free survival (RFS). The column “95% PI” reports the 95% prediction interval for the true effect in a new study. τ²(between) and τ²(within) indicate between-study and within-study variance components, respectively. I²-like(total), I²-like(between), and I²-like(within) denote the proportion of total, between-study, and within-study variability attributable to heterogeneity rather than sampling error. “–” indicates statistics that were not applicable or not estimable (e.g., subgroups with k < 2, where heterogeneity could not be evaluated).

TAN, tumor-associated neutrophils; CSS, cancer-specific survival; OS, overall survival; DFS, disease-free survival; RFS, recurrence-free survival; WTS, whole-tumor section; TN, tumor nest; TS, tumor stroma; IM, invasive margin; HR, hazard ratio; CI, confidence interval; PI, prediction interval; τ², between- and within-study variance components; I²-like, heterogeneity measures analogous to I².

In the multivariate analysis, two studies (four effect sizes; n = 1,209) provided adjusted HRs accounting for clinicopathological variables. The pooled results further confirmed that high TAN infiltration remained an independent favorable prognostic factor for CSS (HR = 0.66; 95% CI, 0.54–0.81; 95% PI, 0.54–0.81; I²-like = 0%; **Table 2B**, **Figure 2B**).

#### 3.3.2 Region-specific analysis: TN, TS, and IM

In the univariate analysis, four studies evaluated TAN infiltration in TN, two in TS, and three in IM. High TAN levels were consistently associated with improved CSS across all regions, with pooled HRs of 0.60 (95% CI, 0.44–0.81) for TN, 0.57 (95% CI, 0.43–0.75) for TS, and 0.57 (95% CI, 0.38–0.86) for IM, accompanied by low heterogeneity (I²-like ≤ 29.5%; **Table 2A**).

In the multivariate analysis, adjusted HRs were available for TN and TS from two studies, whereas no multivariate data were reported for IM. The pooled multivariate results confirmed that high TAN infiltration remained significantly associated with improved CSS in TN (HR = 0.63; 95% CI, 0.47–0.85) and TS (HR = 0.69; 95% CI, 0.52–0.92), with no evidence of heterogeneity (I²-like = 0%; **Table 2B**).

#### 3.3.3 Clinicopathological subgroup analysis

To assess the robustness of the association between high TAN infiltration and CSS across different clinicopathological contexts, subgroup analyses were conducted according to study region, sample size, UICC stage, preoperative treatment status, detection markers, and assessment methods.

In the univariate analysis (**Table 3A**), most subgroups demonstrated a consistent protective effect. All eligible studies were conducted in Euro–American populations, with no combinable data available from Asian cohorts. High TAN infiltration was associated with improved CSS regardless of sample size, although studies with smaller cohorts (≤200 patients) exhibited a relatively stronger protective effect (HR = 0.50; 95% CI, 0.30–0.84). Among studies stratified by UICC stage, only those including stage I–IV patients were eligible for pooling, yielding a significant survival benefit (HR = 0.57; 95% CI, 0.46–0.72). Preoperative treatment did not materially alter the effect estimates, with similar trends observed in both treated (HR = 0.50) and untreated groups (HR = 0.65). Across the available studies, the association remained stable regardless of the assessment method (IHC or H&E) or marker type (e.g., CD66b), with HRs consistently favoring high TAN infiltration (range, 0.56–0.60; I²-like ≤ 47.4%).

Table 3Pooled hazard ratios for TAN infiltration in clinicopathological subgroup analyses.

**Table:** 

(A) Univariate models																
Variable	Subgroup	CSS					OS					DFS				
		k	n	HR (95% CI)	τ²	I²–like	k	n	HR (95% CI)	τ²	I²–like	k	n	HR (95% CI)	τ²	I²–like
Geographic region	Asian	0	0	–	–	–	15	12	0.76 (0.41–1.40)	1.058	86.4	11	8	0.54 (0.27–1.08)	0.915	89.9
	Euro–American	9	5	0.58 (0.48–0.71)	0.014	15.7	8	6	0.75 (0.65–0.87)	0.006	7.2	6	4	1.29 (0.59–2.80)	0.570	90.3
Sample size group	≤200	3	2	0.50 (0.30–0.84)	0.066	37.2	5	5	0.78 (0.36–1.72)	0.330	80.3	5	3	0.62 (0.32–1.19)	0.253	75.0
	>200	6	3	0.61 (0.51–0.74)	0.004	6.5	18	13	0.78 (0.49–1.25)	0.642	83.1	12	9	0.78 (0.38–1.62)	1.169	93.1
Tumor stage (UICC)	I–III	1	1	–	–	–	9	7	0.51 (0.22–1.19)	1.136	83.8	6	4	0.29 (0.11–0.77)	0.841	85.3
	I–IV	8	4	0.57 (0.46–0.72)	0.024	25.3	14	11	0.97 (0.67–1.43)	0.347	80.4	11	8	1.11 (0.70–1.79)	0.420	88.3
Preoperative treatment status	Yes	3	2	0.50 (0.30–0.84)	0.066	37.2	0	0	–	–	–	2	1	1.08 (0.63–1.83)	0	60.3
	No	4	2	0.65 (0.43–0.98)	0.054	43.6	11	9	0.89 (0.50–1.58)	0.708	89.1	7	5	0.66 (0.36–1.21)	0.409	84.0
	NR	2	1	0.59 (0.46–0.75)	0	0.0	12	9	0.69 (0.40–1.17)	0.527	74.1	8	6	0.75 (0.26–2.20)	1.705	94.2
Detection method	IHC	6	3	0.56 (0.38–0.83)	0.075	47.4	19	15	0.79 (0.49–1.27)	0.764	84.5	13	9	0.54 (0.29–0.99)	0.790	88.9
	HE	3	2	0.60 (0.48–0.76)	0	0	3	2	0.68 (0.57–0.81)	0	0	3	2	1.97 (0.56–6.99)	0.709	91.8
	Manual counting	0	0	–	–	–	1	1	–	–	–	0	0	–	–	–
	Gene expression	0	0	–	–	–	0	0	–	–	–	1	1	–	–	–
TAN marker type	CD66b	6	3	0.56 (0.38–0.83)	0.08	47.4	12	9	0.75 (0.33–1.70)	1.725	89.8	10	6	0.48 (0.19–1.17)	1.19	91.9
	CD15	0	0	–	–	–	2	2	0.60 (0.44–0.83)	0	0	1	1	–	–	–
	MPO	0	0	–	–	–	3	2	0.86 (0.65–1.13)	0	0	0	0	–	–	–
	NA	3	2	0.60 (0.48–0.76)	0	0	6	5	0.70 (0.61–0.81)	0	0	6	5	1.26 (0.71–2.23)	0.263	85.8

(B) Multivariate models																
Variable	Subgroup	CSS					OS					DFS				
		k	n	HR (95% CI)	τ²	I²–like	k	n	HR (95% CI)	τ²	I²–like	k	n	HR (95% CI)	τ²	I²–like
Geographic region	Asian	0	0	–	–	–	8	6	0.82 (0.33–2.04)	1.165	85.7	6	4	0.42 (0.14–1.24)	1.016	90.8
	Euro–American	4	2	0.66(0.54–0.81)	0	0	2	1	0.67 (0.56–0.81)	0	0	2	2	1.55 (1.16–2.06)	0	0
Sample size group	≤200	0	0	–	–	–	1	1	–	–	–	0	0	–	–	–
	>200	4	2	0.66(0.54–0.81)	0	0	9	6	0.68 (0.30–1.51)	0.906	84.7	8	6	0.67 (0.27–1.62)	1.053	92.1
Tumor stage (UICC)	I–III	0	0	–	–	–	4	2	0.20 (0.12–0.34)	0	0	4	2	0.17 (0.10–0.27)	0	44.1
	I–IV	4	2	0.66(0.54–0.81)	0	0	6	5	1.26 (0.69–2.29)	0.410	88.7	4	4	1.25 (0.69–2.25)	0.157	86.6
Preoperative treatment status	Yes	2	1	0.64 (0.46–0.91)	0	0	0	0	–	–	–	0	0	–	–	–
	No	0	0	–	–	–	3	3	1.33 (0.58–3.04)	0.240	90.0	2	2	0.95 (0.28–3.18)	0.358	94.0
	NR	2	1	0.67 (0.52–0.87)	0	0	7	4	0.52 (0.17–1.64)	1.210	85.6	6	4	0.56 (0.16–1.97)	1.458	93.1
Detection method	IHC	2	1	0.64 (0.46–0.91)	0	0	8	6	0.82 (0.33–2.04)	1.165	85.7	6	4	0.42 (0.14–1.24)	1.016	90.8
	HE	2	1	0.67 (0.52–0.87)	0	0	2	1	0.67 (0.56–0.81)	0	0	1	1	–	–	–
	Manual counting	0	0	–	–	–	0	0	–	–	–	0	0	–	–	–
	Gene expression	0	0	0	0	0	0	0	0	0	0	1	1	–	–	–
TAN marker type	CD66b	2	1	0.64 (0.46–0.91)	–	–	6	4	0.68 (0.18–2.54)	1.663	87.7	5	3	0.40 (0.09–1.79)	1.555	92.9
CD15	0	0	–	–	–	0	0	–	–	–	1	1	–	–	–
MPO	0	0	–	–	–	1	1	–	–	–	0	0	–	–	–
NA	2	1	0.67 (0.52–0.87)	0	0	3	2	0.65 (0.55–0.77)	0	0	2	2	1.55 (1.16–2.06)	0	0

All pooled estimates were obtained using multilevel random–effects models (rma.mv, REML), with effect sizes nested within studies when multiple cohorts or tumor regions were reported. The column τ² denotes the estimated between–study variance component (or the total random–effects variance when the within–study variance component is zero). I²–like represents the proportion of total variance attributable to between–study heterogeneity and is conceptually analogous to the conventional I² statistic. “–” indicates statistics that were not applicable or not estimable (e.g., subgroups with k < 2, where heterogeneity could not be evaluated). “NR” denotes that the corresponding covariate (e.g., preoperative treatment status) was not reported in the primary studies; “NA” indicates that no specific TAN marker was applied (e.g., H&E–based assessments without an immunohistochemical marker). “CD66b” includes studies using CEACAM8 as an equivalent TAN marker.

TAN, tumor–associated neutrophils; CSS, cancer–specific survival; OS, overall survival; DFS, disease–free survival; HR, hazard ratio; CI, confidence interval; k, number of effect sizes; n, number of studies; NR, not reported; NA, not applicable; IHC, immunohistochemistry; HE, hematoxylin–eosin staining.

For CSS in the multivariate analysis, the number of combinable studies was limited, and some cohorts overlapped. Therefore, meaningful pooled subgroup or stratified analyses could not be performed, and the findings are presented descriptively (**Table 3B**).

### 3.4 Overall survival

#### 3.4.1 Overall analysis (all regions combined)

**Figure 3:**
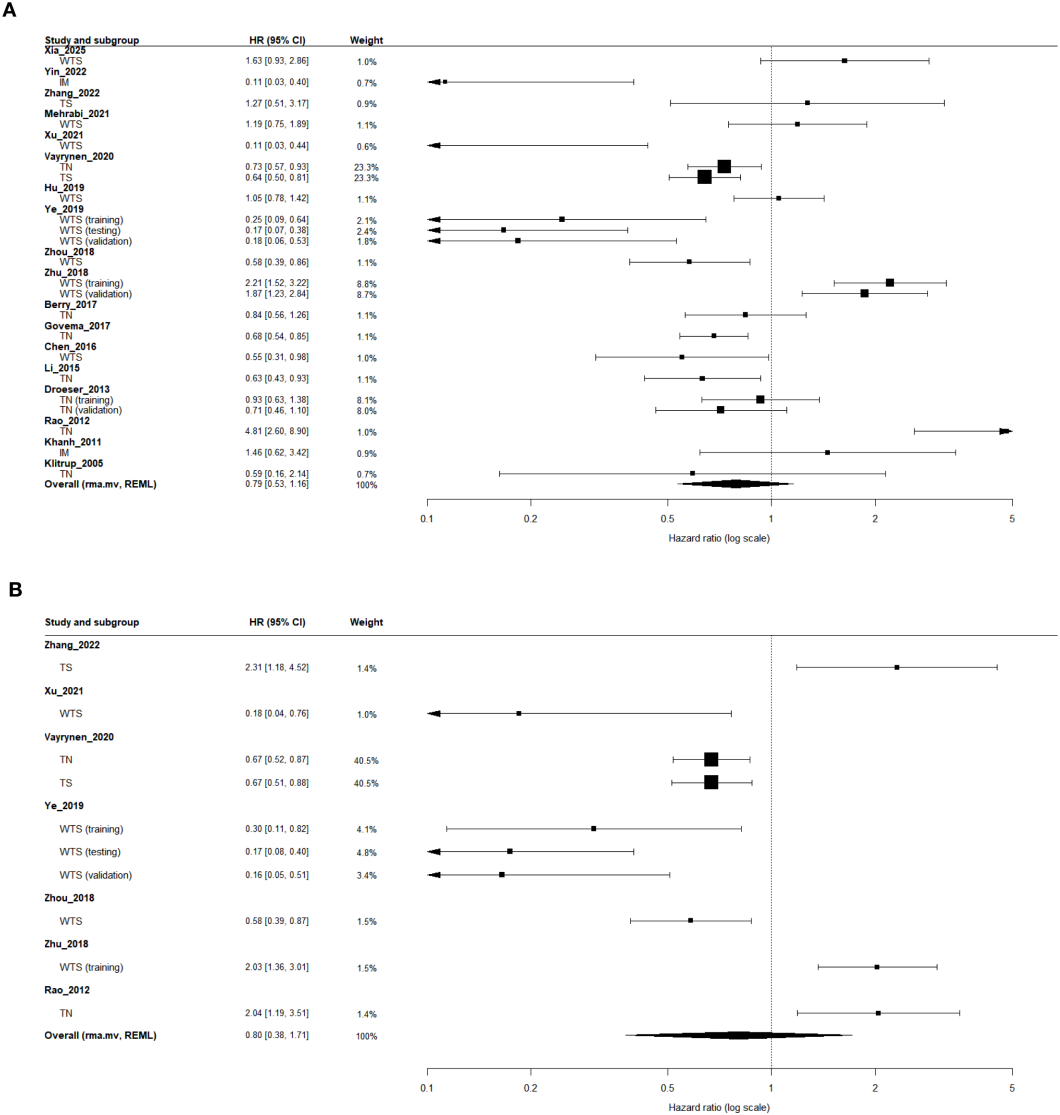
Forest plots of the association between tumor-associated neutrophil (TAN) infiltration and overall survival (OS). **(A)** Univariate analysis. **(B)** Multivariable analysis adjusted for clinicopathological covariates. Region-specific effect sizes (tumor nest [TN], tumor stroma [TS], invasive margin [IM], and whole-tumor section [WTS]) were pooled using a multilevel random-effects model (rma.mv, REML) to account for the within-study correlation of multiple tumor regions and/or cohorts. Hazard ratios (HRs; log scale) < 1 indicate improved survival in patients with high versus low TAN infiltration.

In the univariate analysis (**Table 2A**; **Figure 3A**), 18 independent studies (23 effect sizes; n = 7,856) were included. The pooled estimate from a multilevel random-effects model (rma.mv, REML) showed no significant association between high TAN infiltration and OS (HR = 0.79; 95% CI, 0.53–1.16; 95% PI, 0.17–3.73), with substantial heterogeneity (I²-like = 81.1%).

In the multivariate analysis (**Table 2B**; **Figure 3B**), 7 studies (10 effect sizes; n = 4,000) were eligible. The pooled multivariate results similarly demonstrated no statistically significant association (HR = 0.80; 95% CI, 0.38–1.71; 95% PI, 0.10–6.17; I²-like = 85.4%), indicating that high TAN infiltration did not emerge as an independent prognostic factor for OS after adjustment for clinicopathological covariates.

#### 3.4.2 Region-specific analysis (WTS, TN, TS, and IM)

In the univariate analysis (**Table 2A**), eight studies evaluated TAN infiltration in WTS, seven in TN, two in TS, and two in IM. The pooled estimates indicated no statistically significant associations between TAN levels and OS across any region: WTS (HR = 0.70; 95% CI, 0.37–1.33; I²-like = 84.1%), TN (HR = 0.93; 95% CI, 0.56–1.56; I²-like = 81.8%), TS (HR = 0.78; 95% CI, 0.42–1.42; I²-like = 50.5%), and IM (HR = 0.42; 95% CI, 0.03–5.19; I²-like = 90.8%).

In the multivariate analysis (**Table 2B**), WTS, TN, and TS contributed sufficient data for pooling in multivariate models, and none showed statistically significant associations with OS: WTS (HR = 0.50; 95% CI, 0.16–1.51; I²-like = 83.4%), TN (HR = 1.14; 95% CI, 0.38–3.39; I²-like = 92.4%), and TS (HR = 1.20; 95% CI, 0.36–4.01; I²-like = 91.2%). No multivariate effect sizes were available for IM.

#### 3.4.3 Clinicopathological subgroup analysis

In the univariate analysis (**Table 3A**), statistically significant protective effects were observed in several clinicopathological subgroups. Studies conducted in Euro–American populations reported a significant association between high TAN infiltration and improved OS (HR = 0.75; 95% CI, 0.65–0.87; I²-like = 7.2%), whereas studies from Asia did not show statistical significance (HR = 0.76; 95% CI, 0.41–1.40; I²-like = 86.4%). With respect to detection methods, studies using H&E staining demonstrated a significant survival advantage (HR = 0.68; 95% CI, 0.57–0.81; I²-like = 0%). Among marker-based subgroups, studies employing CD15 (HR = 0.60; 95% CI, 0.44–0.83; I²-like = 0%) and those without reported TAN marker information (HR = 0.70; 95% CI, 0.61–0.81; I²-like = 0%) also showed significant protective effects. Results from other subgroups were directionally consistent but did not reach statistical significance.

In the multivariate analysis (**Table 3B**), high TAN infiltration continued to exhibit significant independent protective effects in several subgroups. Studies from Euro–American populations showed a pronounced survival benefit (HR = 0.67; 95% CI, 0.56–0.81; I²-like = 0%), and the effect was particularly strong among patients with early-stage disease (stage I–III) (HR = 0.20; 95% CI, 0.12–0.34; I²-like = 0%). In addition, studies using H&E staining (HR = 0.67; 95% CI, 0.56–0.81; I²-like = 0%) and those without reported TAN marker information (HR = 0.65; 95% CI, 0.55–0.77; I²-like = 0%) consistently demonstrated significant survival advantages.

### 3.5 Disease-free survival

#### 3.5.1 Overall analysis (all regions combined)

**Figure 4:**
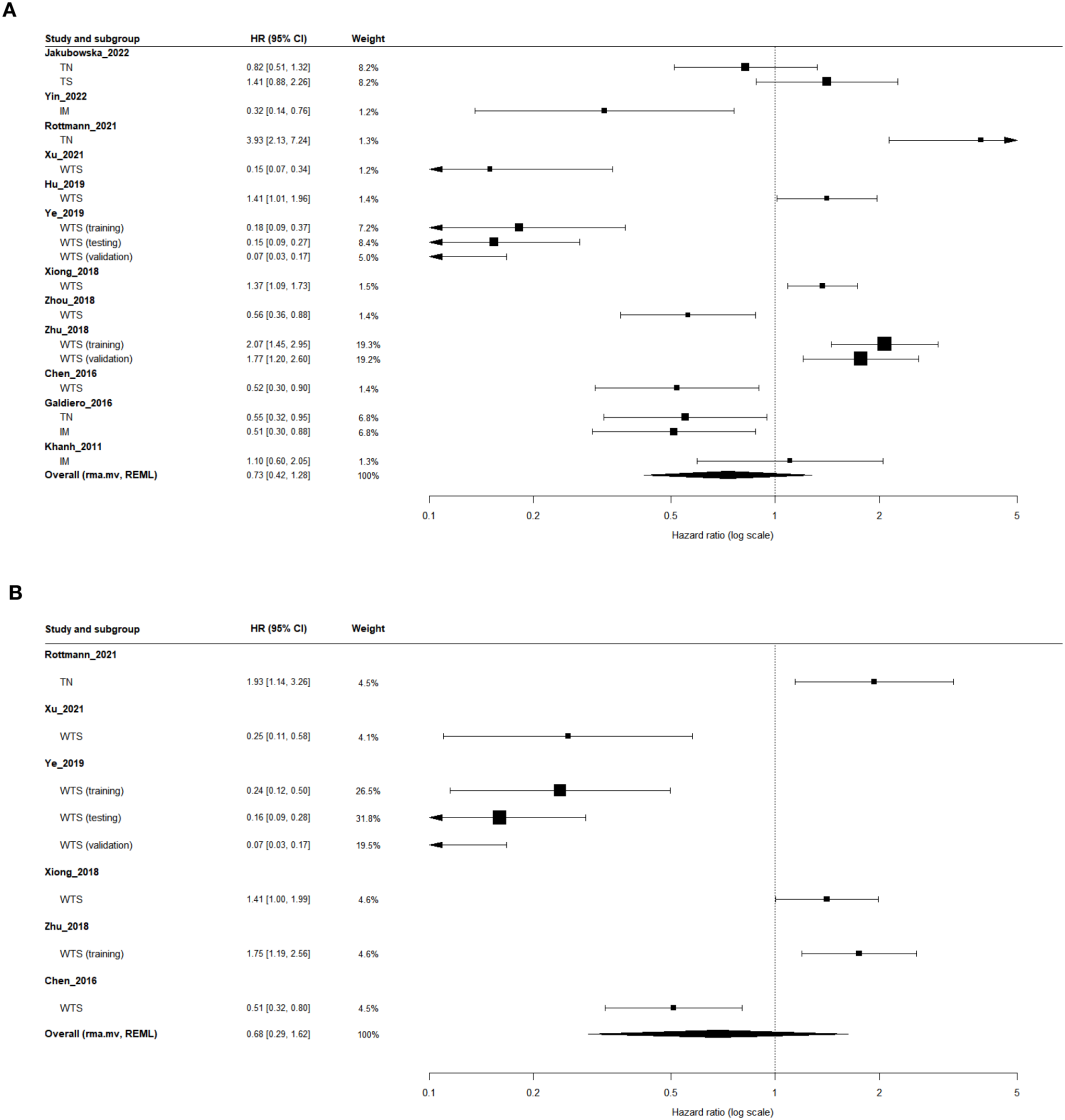
Forest plots of the association between tumor-associated neutrophil (TAN) infiltration and disease-free survival (DFS). **(A)** Univariate analysis. **(B)** Multivariable analysis adjusted for clinicopathological covariates. Region-specific effect sizes (tumor nest [TN], tumor stroma [TS], invasive margin [IM], and whole-tumor section [WTS]) were pooled using a multilevel random-effects model (rma.mv, REML) to account for the within-study correlation of multiple tumor regions and/or cohorts. Hazard ratios (HRs; log scale) < 1 indicate improved survival in patients with high versus low TAN infiltration.

In the univariate analysis (**Table 2A**; **Figure 4A**), 12 independent studies (17 effect sizes; n = 5,521) were included. The pooled estimate from the multilevel random-effects model (rma.mv, REML) showed no significant association between high TAN infiltration and DFS (HR = 0.73; 95% CI, 0.42–1.28; 95% PI, 0.10–5.14), with substantial between-study heterogeneity (I²-like = 91.1%).

In the multivariate analysis (**Table 2B**; **Figure 4B**), six studies (eight effect sizes; n = 4,025) were eligible. Consistent with the univariate findings, the pooled estimate did not reach statistical significance (HR = 0.67; 95% CI, 0.27–1.62; 95% PI, 0.07–6.70; I²-like = 92.1%), indicating that high TAN infiltration did not emerge as an independent prognostic factor for DFS after adjustment for clinicopathological covariates.

#### 3.5.2 Region-specific analysis (WTS, TN, TS, and IM)

In the univariate analysis (**Table 2A**), seven studies evaluated TAN infiltration in WTS, three in TN, one in TS, and three in IM. The pooled estimates showed no statistically significant associations between TAN levels and DFS in any region: WTS (HR = 0.60; 95% CI, 0.27–1.32; I²-like = 92.7%), TN (HR = 1.20; 95% CI, 0.37–3.84; I²-like = 92.7%), and IM (HR = 0.59; 95% CI, 0.30–1.16; I²-like = 66.4%). The TS group contained only one study and was therefore not pooled.

In the multivariate analysis (**Table 2B**), only the WTS group provided sufficient data for pooling (seven effect sizes). The pooled multivariate results showed no statistically significant association (HR = 0.54; 95% CI, 0.21–1.41; 95% PI, 0.05–5.51; I²-like = 91.7%).

To further assess the robustness of the DFS-related findings with respect to endpoint definition, analyses were repeated after restricting the dataset to studies reporting DFS only. The pooled HRs across overall and region-specific models were largely consistent with those from the combined DFS/RFS analyses, with overlapping confidence intervals and consistent effect directions (**Supplementary Table 5**).

#### 3.5.3 Clinicopathological subgroup analysis

In the univariate analysis (**Table 3A**), substantial variability was observed across clinicopathological subgroups, with overall heterogeneity being high (I²-like ranging from ~75% to 94%). Statistically significant protective effects were identified in two subgroups: patients with UICC stage I–III disease (HR = 0.29; 95% CI, 0.11–0.77; I²-like = 85.3%) and studies using IHC-based TAN assessment (HR = 0.54; 95% CI, 0.29–0.99; I²-like = 88.9%). Other subgroups showed directionally similar trends but did not reach statistical significance.

In the multivariate analysis (**Table 3B**), a statistically significant protective effect persisted only in patients with UICC stage I–III disease (HR = 0.17; 95% CI, 0.10–0.27; I²-like = 44.1%). In contrast, studies conducted in Euro–American populations (HR = 1.55; 95% CI, 1.16–2.06; I²-like = 0%) and those without reported TAN marker information (HR = 1.55; 95% CI, 1.16–2.06; I²-like = 0%) showed significantly increased recurrence risk associated with high TAN infiltration. No other subgroup demonstrated statistically significant associations.

### 3.6 Publication bias and small-study effect analysis

In assessing potential publication bias and small-study effects, Egger’s regression test, Begg’s rank correlation test, and the trim-and-fill method were applied across six analytical sets corresponding to CSS, OS, and DFS in both univariate and multivariate models (**Table 4**). Overall, most tests yielded non-significant results (P > 0.10), indicating no clear evidence of publication bias. For example, in the CSS–uni model, the pooled HR was 0.61 (95% CI, 0.48–0.77), and the trim-and-fill procedure did not impute missing studies (k_0_; = 0), yielding an identical adjusted estimate and a symmetrical funnel plot.

**Table 4:** Publication bias and small-study effect tests (Egger, Begg, and trim-and-fill).

Outcome	Model	k	HR	95% CI	Egger p	Begg p	Trim-and-Fill (k_0_)	Interpretation
CSS	Univariate	5	0.61	0.48–0.77	0.718	0.817	0	No evidence of publication bias; Egger and Begg tests not significant (k < 10).
CSS	Multivariate	2	NA	NA	NA	NA	NA	Too few studies for formal bias assessment (k < 3).
OS	Univariate	18	0.83	0.57–1.19	0.034	0.454	0	Egger test significant; possible mild asymmetry, but no imputed studies.
OS	Multivariate	7	0.89	0.45–1.73	0.135	1.000	0	No significant bias detected; limited power due to small k (k < 10).
DFS	Univariate	12	0.75	0.44–1.27	0.011	0.116	0	Egger test significant; mild asymmetry with limited impact on pooled HR.
DFS	Multivariate	6	0.76	0.36–1.61	0.008	0.272	0	Egger test significant but very small k; likely false-positive signal.

“NA” indicates that formal publication bias testing was not applicable (e.g., when the number of studies was too small, k < 3). Egger and Begg tests have low statistical power in small meta-analyses (typically k < 10); results for such models should therefore be interpreted with caution. The trim-and-fill procedure did not impute any potentially missing studies in any model (k_0_ = 0 for all analyses).

CSS, cancer-specific survival; OS, overall survival; DFS, disease-free survival; HR, hazard ratio; CI, confidence interval; k, number of studies; k_0_, number of imputed studies in the trim-and-fill analysis; NA, not applicable.

In the OS–uni model, Egger’s test suggested mild asymmetry (P = 0.034), whereas Begg’s test remained non-significant (P = 0.454), and trim-and-fill still imputed no additional studies (k_0_ = 0), implying that any potential small-study effects had limited influence on the overall estimate. A similar pattern was observed in the DFS–uni model, where Egger’s test indicated modest asymmetry (P = 0.011), but no studies were added by trim-and-fill and the adjusted HR (0.75; 95% CI, 0.44–1.27) remained consistent with the original estimate. For multivariate models (CSS–multi, OS–multi, DFS–multi), Begg’s tests were non-significant and trim-and-fill did not impute additional studies (all k_0_ = 0); although Egger’s test yielded a nominally significant P value for DFS–multi (P = 0.008), the absence of imputed studies and the similarity between original and adjusted estimates suggest that any small-study effects are unlikely to materially bias the results. Notably, because several models included fewer than ten studies (k < 10), the statistical power of Egger’s and Begg’s tests was limited, and these findings should therefore be interpreted with caution.

Taken together, no substantial publication bias or small-study effects were detected, supporting the credibility and stability of the pooled effect estimates.

### 3.7 Sensitivity analysis

To verify the robustness of the main findings, sensitivity analyses were performed across all six outcome models (**Supplementary Table 6**). Leave-one-out analyses showed minimal changes in pooled HRs (absolute difference < 0.15) with consistent effect directions, indicating that no single study materially influenced the overall estimates. Influence diagnostics (Cook’s D and DFBETAS) did not identify high-leverage or influential outlier studies, and all models exhibited stable convergence during weighted least-squares refitting, suggesting that the results were not driven by aberrant observations.

Robust standard error (robust SE) adjustments produced central HRs that were highly consistent with the primary analyses (differences < 5%), with only modest widening of confidence intervals, indicating low sensitivity to variance assumptions. Directed exclusion strategies based on study quality, sample size, detection method, and TAN markers further confirmed the stability of the results: pooled HRs remained within 0.46–0.66 after removing low-quality or small-sample studies, and stronger protective effects were observed in analyses restricted to IHC-based assessments or CD66b markers (HR ≈ 0.27–0.50).

Overall, the sensitivity analyses demonstrated consistent robustness across all outcome models. High TAN infiltration showed stable and significant improvement in CSS (HR ≈ 0.58–0.66; I²-like ≈ 0%) in both univariate and multivariate analyses, while OS and DFS exhibited concordant protective trends (HR ≈ 0.46–0.73) without effect reversal. These results collectively support the reliability and statistical stability of the association between high TAN infiltration and favorable survival outcomes.

### 3.8 Exploration of heterogeneity and meta-regression analysis

Given the low heterogeneity observed in CSS models, our exploration of heterogeneity focused on OS and DFS. In the OS univariate model, overall between-study heterogeneity was substantial (τ² = 0.592; I²-like = 81.1%). Subgroup analyses indicated that study region and tumor stage were major drivers of this variability. Among non-Asian (Euro–American) studies, heterogeneity markedly decreased (τ² = 0.0064; I²-like = 7.2%), and in the multivariate OS model, heterogeneity in Euro–American cohorts was essentially abolished (τ² = 0; I²-like = 0%). Similarly, in the multivariate OS model stratified by UICC stage, the stage I–III subgroup showed no residual heterogeneity (τ² = 0), whereas studies including stage I–IV disease retained high heterogeneity (τ² = 0.4099; I²-like = 88.7%), suggesting that differences in stage composition may underlie inconsistent effect estimates. For DFS, overall heterogeneity was likewise high (τ² = 1.0533; I²-like = 92.1%), and subgroup patterns by region and stage paralleled those observed for OS. Detection method (IHC vs H&E) modestly attenuated heterogeneity in selected models, whereas sample size and preoperative treatment had relatively minor impact.

On this basis, multilevel meta-regression analyses were conducted to quantify the contribution of key moderators to between-study variance (**Supplementary Table 7**). In the OS univariate model, none of the covariates reached conventional statistical significance, although UICC stage showed a positive trend (β = 0.62; P = 0.142), which remained directionally consistent after CR2-robust variance correction (p_CR2 = 0.176). In contrast, the multivariate OS model showed a marked reduction of heterogeneity after inclusion of covariates (τ² decreasing from 0.933 to approximately 0), and the overall regression was highly significant (QM = 75.67; P < 0.001). UICC stage (β = 2.31; 95% CI, 1.70–2.91; P < 0.001) and TAN detection marker (β = −1.25; 95% CI, −1.77 to −0.73; P < 0.001) emerged as the principal explanatory variables, while smaller sample size (≤200 patients) was also associated with lower HRs (β = −1.38; P = 0.001). After CR2 correction, stage and marker remained statistically significant (p_CR2 < 0.01), indicating that these two factors robustly account for nearly all between-study heterogeneity in OS; the extremely small CR2 p-value for sample size likely reflects numerical instability rather than a genuinely stronger effect.

For DFS in the univariate setting, several covariates significantly explained heterogeneity: UICC stage (β = −1.23; P = 0.0025), detection marker (β = −0.96; P = 0.021), and detection method (β = −0.61; P = 0.048), suggesting that differences in staging and assessment strategies may yield divergent effect directions. After CR2 adjustment, the associations for UICC stage and detection marker were attenuated and became non-significant (p_CR2 ≥ 0.27), although the directions of effect remained unchanged; CR2-based tests were not available for detection method owing to convergence limitations. In the multivariate DFS model, only UICC stage remained significant (β = 2.01; 95% CI, 1.14–2.87; P < 10^-5^), explaining approximately 90% of the between-study variance (τ² reduced from 1.053 to 0.045). However, because the comprehensive multivariate model failed to converge stably in the presence of multiple correlated moderators, CR2-robust testing could not be reliably applied.

Taken together, subgroup and meta-regression analyses yielded highly consistent patterns: tumor stage and TAN detection markers are the predominant sources of heterogeneity in OS and DFS models, whereas detection method, sample size, and preoperative treatment exert comparatively modest influence. The staging variable remained consistently significant or directionally stable across multiple models, suggesting that differences in tumor stage distribution are a key determinant of variability in the prognostic impact of TAN infiltration.

## 4 Discussion

With the increasing depth of research into CRC, numerous immune-related biomarkers have been shown to carry important prognostic information, providing critical clues for improving treatment response and optimizing therapeutic strategies (7). However, the prognostic role of TANs in CRC has remained controversial, with some studies suggesting a survival benefit and others indicating a potential tumor-promoting effect (7, 8, 16, 18). In this study, we conducted an up-to-date systematic review and multilevel random-effects meta-analysis integrating three key survival endpoints (CSS, OS, and DFS), jointly modeling effect sizes derived from different tumor regions (TN, TS, IM, and WTS), and systematically evaluating the influence of tumor region, sample size, tumor stage, TAN markers, and detection methods. Our findings indicate that TAN infiltration in CRC is overall associated with a predominantly protective prognostic association, with the most robust and consistent evidence observed for CSS, whereas the associations with OS and DFS appeared more variable across studies.

In the CSS models, we observed the most consistent and robust evidence. In both univariate and multivariate analyses, high TAN infiltration was significantly associated with a reduced risk of CRC-specific mortality, with pooled hazard ratios around 0.6, low between-study heterogeneity, and no indication of instability in sensitivity or publication bias analyses. Region-specific analyses further demonstrated that, in studies with available regional data, the protective association between high TAN density and CSS was highly consistent across tumor nest, tumor stroma, and invasive margin. By contrast, in the OS and DFS models, although a similar trend toward favorable prognosis was observed, the overall pooled effects did not reach statistical significance and were accompanied by substantial heterogeneity.

Subgroup analyses and multilevel meta-regression provided important insights into the sources of this variability. For OS, high TAN infiltration was associated with reduced mortality predominantly in Euro–American cohorts, whereas studies conducted in Asian populations showed more heterogeneous or non-significant effects. The protective association was particularly evident in patients with UICC stage I–III disease, while it was clearly attenuated in cohorts including stage IV cases. A similar pattern was observed for DFS: studies focusing on earlier-stage disease and those employing immunohistochemistry-based TAN assessment tended to demonstrate a reduced risk of recurrence, whereas cohorts enriched for advanced-stage disease or lacking clearly defined marker information showed attenuated or even reversed associations. In more granular stratifications, the protective effect was weakened in studies including stage I–IV patients, and in studies without explicit reporting of TAN markers, high TAN density was even linked to a higher risk of recurrence. Multilevel meta-regression further identified tumor stage as the principal covariate explaining heterogeneity in OS and DFS, with TAN markers acting as an additional important modifying factor, while sample size and detection method contributed to heterogeneity in selected models but accounted for comparatively less between-study variance.

An additional source of heterogeneity in the OS and DFS models was the type of TAN marker, indicating that commonly used TAN markers are not biologically interchangeable. Although CD66b, MPO, CD15, CD177, CD10, H&E-based morphology, and transcriptomic deconvolution were aggregated under a unified operational definition of tumor-associated neutrophils for meta-analytic feasibility, these approaches capture distinct neutrophil subsets, activation states, and inflammatory contexts within the tumor microenvironment. Specifically, CD66b and CD10 preferentially label mature and activated neutrophils, whereas MPO reflects oxidative and cytotoxic activity, and CD15 lacks neutrophil specificity and may include broader myeloid populations. H&E-based assessments integrate multiple immune components and represent the overall inflammatory reaction rather than neutrophil-specific biology, while transcriptomic estimates may encompass heterogeneous granulocytic or myeloid-derived suppressor–like populations, particularly in advanced disease. These biological differences provide a plausible explanation for why marker type substantially contributed to heterogeneity in OS and DFS, with a comparatively limited impact on CSS.

Beyond marker-related heterogeneity, the pronounced variability observed in OS and DFS also reflects the stage-dependent and phenotypically plastic nature of TAN biology. Our meta-regression demonstrated that UICC stage was the most important determinant of heterogeneity in these endpoints: in patients with stage I–III disease, high TAN infiltration was associated with markedly reduced risks of death and recurrence, whereas in cohorts including stage IV patients, this effect was attenuated or even reversed. These findings are consistent with experimental evidence indicating that TANs can undergo a dynamic transition from antitumor to protumor phenotypes during tumor progression. Early tumor–derived neutrophil subsets such as CD11b^+^CD15^high^CD10^-^CD16^low^ cells exhibit more pronounced antitumor features, including enhanced immune surveillance and cytotoxic capacity, with higher expression of ICAM-1 and CD95 in mesenteric tissues (15, 47). As tumors progress, soluble factors released by cancer and stromal cells can drive TAN polarization from a pro-immunogenic N1 phenotype toward an immunosuppressive N2 phenotype, characterized by promotion of angiogenesis, epithelial–mesenchymal transition, and immune evasion (48). Animal models likewise suggest that neutrophils may suppress tumor growth and invasion in early stages but facilitate metastatic dissemination in later stages (49). The patterns of statistical heterogeneity observed in our analysis are therefore highly consistent with this stage-dependent, plastic nature of TAN biology.

Beyond biological heterogeneity, intrinsic differences among survival endpoints further contribute to the distinct patterns observed for CSS, OS, and DFS. CSS specifically captures tumor-related mortality and is less sensitive to non-cancer–related competing risks, thereby more directly reflecting intrinsic tumor biology, particularly in older patients or those with multiple comorbidities. In contrast, OS is strongly influenced by competing risks such as cardiovascular events and infections, which may dilute cancer-specific effects. DFS captures both local recurrence and distant metastasis but is also affected by postoperative inflammation, tissue remodeling, follow-up duration, and variability in endpoint definitions, especially in studies enriched for early-stage disease or heterogeneous treatment strategies. These characteristics likely contribute to the greater statistical stability observed for CSS compared with OS and DFS in our meta-analysis.

In sensitivity analyses, the study by Ye et al. (2019) was identified as a high-influence study with a hazard ratio lower than the overall mean but a concordant direction of effect (30). This study included a relatively large sample size, used immunohistochemical assessment of CD66b-positive TANs, and validated its findings across three independent cohorts (training, testing, and validation). Its sampling strategy was relatively extensive and approximated whole-tumor assessment, which may account for the stronger protective effect observed. Importantly, exclusion or separate modeling of this study did not materially alter the overall pooled estimates, indicating that it contributed high-weight, concordant evidence rather than acting as a source of bias.

This study has several strengths. Based on our current systematic search, it represents, to our knowledge, the first multilevel random-effects meta-analysis in the past decade to quantitatively evaluate the prognostic significance of TANs in CRC. By explicitly modeling effect sizes nested within studies, our approach accounts for within-study correlations arising from multiple tumor regions or cohorts and provides more granular and methodologically rigorous quantitative evidence. We further demonstrated that the protective association between TAN infiltration and CSS was highly consistent across tumor regions (TN, TS, and IM), and identified tumor stage and TAN marker type as key effect modifiers through subgroup analyses and multilevel meta-regression. The study was conducted under PRISMA guidance with comprehensive multi-database searching and transparent reporting, enhancing reproducibility and interpretability.

Several limitations should also be acknowledged. Some primary studies lacked detailed baseline information, such as preoperative treatment, comorbidity profiles, and full stage distributions, which may have introduced residual confounding. TAN assessment methods and cut-off definitions were heterogeneous, limiting direct comparability across studies. A proportion of hazard ratios had to be reconstructed from Kaplan–Meier curves; although this approach has been validated and associated error is generally considered acceptable, minor estimation bias cannot be excluded. In addition, restriction to English-language full-text publications and limited numbers of studies in certain subgroups reduced statistical power for some publication bias assessments, and these results should therefore be interpreted with caution.

Despite these limitations, our findings have important implications in the context of evolving CRC treatment strategies. Current management has shifted from surgery alone to multimodal regimens incorporating chemotherapy, targeted therapy, and immune checkpoint inhibitors (50). However, in the predominantly microsatellite-stable population, clinical benefit from immunotherapy remains limited, and tumors often display an immunologically “cold” phenotype (51). Previous work suggests that remodeling the tumor microenvironment and enhancing innate immune cell infiltration may be key to improving therapeutic response (52). The functional plasticity of TANs therefore offers a potential window for therapeutic intervention: by modulating chemokine axes or polarization signals to bias TANs toward an antitumor phenotype, it may be possible to convert immunologically “cold” tumors into more responsive states (48, 49). Future studies integrating spatial transcriptomics and single-cell multi-omics will be essential to refine phenotypically resolved TAN assessment and to establish a stronger biological foundation for incorporating TAN-related metrics into individualized prognostic and therapeutic strategies for CRC.

## Data Availability

The original contributions presented in the study are included in the article/**Supplementary Material**. Further inquiries can be directed to the corresponding author.
