# Supplementary material for: Tumor-associated neutrophils and survival outcomes in colorectal cancer: a systematic review and multilevel meta-analysis
Source: Front Oncol. 2026 Feb 25;16:1788605. doi: 10.3389/fonc.2026.1788605 (PMC12975556; doi:10.3389/fonc.2026.1788605)
Supplement: Supplementary file 2 [file Table2.doc]

**Supplementary Table S2. Search strategy based on the Population-Intervention-Comparator-Outcome (PICO) framework.**

| **Population (P)** | **AND** | **Intervention (I)** | **AND** | **Comparator (C)** | **AND** | **Outcome (O)** |
| --- | --- | --- | --- | --- | --- | --- |
| Colorectal neoplasm  OR  Colorectal cancer  OR  Colon cancer  OR  Colonic neoplasm  OR  Rectal cancer  OR  Rectal neoplasm  OR  Colorectal adenocarcinoma  OR  Colorectal cancer cells  OR  CRC |  | Neutrophils  OR  Tumor-infiltrating neutrophils  OR  Tumor-associated neutrophils  OR  Tumor-infiltrating leukocytes  OR  Tumor-associated leukocytes  OR  TAN  OR  TIN |  | None |  | Prognosis  OR  Prognostic  OR  Prediction  OR  Overall survival  OR  Cancer-specific survival  OR  Disease-free survival  OR  Recurrence-free survival  OR  Survival  OR  Clinical outcome |

**Supplementary Table 3. Database-specific search strategies.**

| **Database** | **Search strategy** | | |
| --- | --- | --- | --- |
| Pubmed | #1 | | "Neutrophils"[MeSH] |
|  | #2 | | "Neutrophils"[MeSH] OR "Tumor-infiltrating neutrophils [Title/Abstract]" OR "Tumor-associated neutrophils [Title/Abstract]" OR "Tumor-infiltrating leukocytes [Title/Abstract]" OR "Tumor-associated leukocytes [Title/Abstract]" OR "TAN [Title/Abstract]" OR "TIN [Title/Abstract]" |
|  | #3 | | "Colorectal neoplasms"[MeSH] |
|  | #4 | | "Colorectal neoplasms"[MeSH] OR "Neoplasm, Colorectal [Title/Abstract]" OR "Neoplasms, Colorectal [Title/Abstract]" OR "Colorectal Tumors [Title/Abstract]" OR "Tumor, Colorectal [Title/Abstract]" OR "Colorectal Cancer [Title/Abstract]" OR "CRC" |
|  | #5 | "Prognosis"[MeSH] | |
|  | #6 | "Prognostic"[MeSH] OR "Prognostic [Title/Abstract]" OR "Prediction [Title/Abstract]" OR "survival [Title/Abstract]" OR "Clinical outcome [Title/Abstract]" | |
|  | #7 | #2 AND #4 AND #6 | |
| Embase | #1 | 'neutrophils'/exp | |
|  | #2 | 'tumor-infiltrating neutrophils':ti, ab, kw OR 'tumor-associated neutrophils':ti, ab, kw OR 'tumor-infiltrating leukocytes':ti, ab, kw OR 'tumor-associated leukocytes':ti, ab, kw OR 'TAN':ti, ab, kw OR 'TIN':ti, ab, kw | |
|  | #3 | #1 OR #2 | |
|  | #4 | 'colorectal tumor'/exp | |
|  | #5 | 'colon tumor':ti, ab, kw OR 'colorectal adenoma':ti, ab, kw OR 'colorectal cancer':ti, ab, kw OR 'rectum tumor':ti, ab, kw OR 'CRC':ti, ab, kw | |
|  | #6 | #4 OR #5 | |
|  | #7 | 'prognosis'/exp | |
|  | #8 | 'prognostic':ti, ab, kw OR 'prediction':ti, ab, kw OR 'survival':ti, ab, kw OR 'clinical outcome':ti, ab, kw | |
|  | #9 | #7 OR #8 | |
|  | #10 | #3 AND #6 AND #9 | |
| Web of Science | #1 | Topic: [Neutrophils] | |
|  | #2 | "Tumor-infiltrating neutrophils" OR "Tumor-associated neutrophils " OR "Tumor-infiltrating leukocytes" OR "Tumor-associated leukocytes " OR "TAN" OR "TIN" | |
|  | #3 | #1 OR #2 | |
|  | #4 | Topic: [Colorectal neoplasms] | |
|  | #5 | "Neoplasm, Colorectal " OR "Neoplasms, Colorectal" OR "Colorectal Tumors " OR "Tumor, Colorectal " OR "Colorectal Cancer " OR "CRC" | |
|  | #6 | #4 OR #5 | |
|  | #7 | Topic: [Prognostic] | |
|  | #8 | "Prognostic" OR "Prediction" OR "survival " OR "Clinical outcome " | |
|  | #9 | #7 OR #8 | |
|  | #10 | #3 AND #6 AND #9 | |

**Supplementary Table 4. Definitions of survival indicators.**

| **Survival indicator** | **Definition** |
| --- | --- |
| Cancer-specific survival (CSS) | The time from the diagnosis of colorectal cancer to death attributable specifically to colorectal cancer, or to the last follow-up if the patient is alive or has died from another cause. |
| Overall survival (OS) | The time from the diagnosis of colorectal cancer to death from any cause, or to the last follow-up for surviving patients. |
| Disease-free survival (DFS) | The time from surgery (or randomization) to the first occurrence of any of the following events: tumor recurrence (local or distant), ipsilateral or contralateral second primary cancer, distant metastasis, disease progression, or death from any cause, whichever occurs first. |
| Recurrence-free survival (RFS) | The time from surgery (or randomization) to the first documented tumor recurrence—local, regional, or distant—or death from any cause, whichever occurs first. |

Note: DFS and RFS were treated as closely related time-to-event endpoints for meta-analysis because they both reflect disease control after curative-intent treatment (or randomization) and were defined with substantial overlap across the included studies. This operational grouping aligns with common usage in oncology research and with the National Cancer Institute (NCI) Cancer Dictionary, where DFS is sometimes described using terms such as relapse-free or recurrence-free survival.

**Supplementary Table S5. Comparison of pooled DFS-related HRs based on different endpoint definitions (combined DFS/RFS versus DFS-only).**

| **Tumor region** | **Endpoint definition** | **No. of effects** | **No. of studies** | **Pooled HR** | **95% CI** | **p-value** |
| --- | --- | --- | --- | --- | --- | --- |
| Overall | DFS+RFS | 17 | 12 | 0.73 | 0.42–1.28 | 0.272 |
|  | DFS-only | 15 | 10 | 0.60 | 0.34–1.06 | 0.077 |
| WTS | DFS+RFS | 10 | 7 | 0.60 | 0.27–1.32 | 0.202 |
|  | DFS-only | 10 | 7 | 0.60 | 0.27–1.32 | 0.202 |
| TN | DFS+RFS | 3 | 3 | 1.20 | 0.37–3.84 | 0.760 |
|  | DFS-only | 2 | 2 | 0.69 | 0.47–1.01 | 0.059 |
| TS | DFS+RFS | 1 | 1 | — | — | — |
|  | DFS-only | 1 | 1 | — | — | — |
| IM | DFS+RFS | 3 | 3 | 0.59 | 0.30–1.16 | 0.125 |
|  | DFS-only | 2 | 2 | 0.45 | 0.28–0.71 | <0.001 |

**Note**：HRs were estimated using multilevel random-effects models (rma.mv, REML). HR < 1 indicates a more favorable DFS-related outcome (DFS or RFS, depending on endpoint definition) associated with higher TAN infiltration. “-” indicates that pooling was not performed because fewer than two effect sizes were available.
