# Supplementary material for: Tumor-associated neutrophils and survival outcomes in colorectal cancer: a systematic review and multilevel meta-analysis
Source: Front Oncol. 2026 Feb 25;16:1788605. doi: 10.3389/fonc.2026.1788605 (PMC12975556; doi:10.3389/fonc.2026.1788605)
Supplement: Supplementary file 3 [file Table3.docx]

# Table S6. Sensitivity analyses across six outcome models (CSS, OS, DFS; univariate and multivariate).

| **Outcome** | **Model** | **k** | **Pooled HR**  **(95% CI)** | **I²-like (%)** | **HR range (Leave-one-out)** | **Max Cook's D** | **Robust HR**  **(95% CI)** | **Directional (IHC)** | **Directional (CD66b)** | **Comment** |
| --- | --- | --- | --- | --- | --- | --- | --- | --- | --- | --- |
| CSS | Univariate | 9 | 0.58 (0.50–0.69) | 0.3 | 0.55–0.62 | 0.04 | 0.58 (0.49–0.69) | 0.57 (0.44–0.74) | 0.57 (0.44–0.74) | Significant, low heterogeneity |
| CSS | Multivariate | 4 | 0.66 (0.54–0.81) | 0.0 | 0.64–0.67 | 0.03 | 0.66 (0.64–0.69) | NA | NA | Consistent, no influence points |
| OS | Univariate | 10 | 0.63 (0.34–1.18) | 84.5 | 0.55–0.70 | 0.28 | 0.63 (0.28–1.43) | 0.60 (0.27–1.37) | NA | Stable trend, high heterogeneity |
| OS | Multivariate | 7 | 0.63 (0.34–1.18) | 84.5 | 0.55–0.70 | 0.18 | 0.63 (0.28–1.43) | 0.60 (0.27–1.37) | NA | Same direction after robust adjustment |
| DFS | Univariate | 12 | 0.64 (0.38–1.06) | 92.2 | 0.57–0.70 | 0.28 | 0.64 (0.31–1.31) | 0.48 (0.27–0.85) | 0.37 (0.17–0.80) | Protective trend; high heterogeneity |
| DFS | Multivariate | 6 | 0.46 (0.20–1.09) | 93.2 | 0.38–0.95 | 0.49 | 0.46 (0.15–1.45) | 0.30 (0.12–0.74) | 0.27 (0.09–0.78) | Most pronounced protective effect in CD66b subset |

**Note**:Sensitivity analyses across six outcome–model combinations (CSS, OS, DFS; univariate and multivariate models) are presented, including leave-one-out re-estimation of the pooled HR (HR range), influence diagnostics (maximum Cook’s D and DFBETAS), CR2-robust SE adjustment (“Robust HR”), and rule-based directional exclusion restricted to IHC-based assessments or CD66b markers. “NA” indicates that the corresponding analysis was not applicable or not performed (e.g., because of insufficient data or lack of IHC/CD66b subsets).

**Abbreviations**: CSS, cancer-specific survival; OS, overall survival; DFS, disease-free survival; HR, hazard ratio; CI, confidence interval; I²-like, heterogeneity measure analogous to I²; k, number of studies; SE, standard error; Cook’s D, Cook’s distance; DFBETAS, standardized difference in regression coefficients; IHC, immunohistochemistry; CD66b (CEACAM8), carcinoembryonic antigen-related cell adhesion molecule 8; NA, not applicable.
