# Supplementary material for: Tumor-associated neutrophils and survival outcomes in colorectal cancer: a systematic review and multilevel meta-analysis
Source: Front Oncol. 2026 Feb 25;16:1788605. doi: 10.3389/fonc.2026.1788605 (PMC12975556; doi:10.3389/fonc.2026.1788605)
Supplement: Supplementary file 4 [file Table4.docx]

**Table S7. Multilevel meta-regression analyses of potential sources of heterogeneity in OS and DFS models.**

| **Outcome** | **Model** | **Moderator** | **β** | **95% CI** | **p** | **τ²_before** | **τ²_after** | **p_CR2** | **Interpretation** |
| --- | --- | --- | --- | --- | --- | --- | --- | --- | --- |
| OS‑uni | Univariate | UICC Stage (I–IV) | 0.62 | –0.20 – 1.39 | 0.142 | 0.592 | 0.575 | 0.176 | Positive trend; not significant under CR2 |
| OS‑uni | Univariate | Pre‑op treatment | –0.27 | –1.06 – 0.53 | 0.506 | 0.592 | 0.620 | 0.512 | No effect |
| OS‑multi | Multivariate | UICC Stage (I–IV) | 2.31 | 1.70 – 2.91 | <0.001 | 0.933 | ≈0 (3.44×10⁻¹²) | 0.00089 | Significant positive effect |
| OS‑multi | Multivariate | Marker (Others) | –1.25 | –1.77 – 0.73 | <0.001 | 0.933 | ≈0 (3.44×10⁻¹²) | 0.00143 | Significant negative effect |
| OS‑multi | Multivariate | Sample size >200 | –1.38 | –2.16 – 0.60 | 0.001 | 0.933 | ≈0 (3.44×10⁻¹²) | 4.41E‑20* | Likely numerical artifact |
| DFS‑uni | Univariate | UICC Stage (I–IV) | –1.23 | –2.03 – 0.43 | 0.0025 | 1.053 | 0.542 | 0.272 | Main driver; marginal under CR2 |
| DFS‑uni | Univariate | Detection marker | –0.96 | –1.77 – 0.15 | 0.021 | 1.053 | 0.767 | 1.000 | Significant in univariate; null under CR2 |
| DFS‑uni | Univariate | Detection method | –0.61 | –1.22 – 0.00 | 0.048 | 1.053 | 0.788 | NA | Significant in univariate; weak overall |
| DFS‑multi | Multivariate | UICC Stage (I–IV) | 2.01 | 1.14 – 2.87 | <1E‑5 | 1.053 | 0.045 | NA | Model converged in univariate only |
| DFS‑multi | Multivariate | Comprehensive model | NA | NA | NA | 1.053 | NA | NA | Model failed; CR2 skipped |

**Note**: Multilevel meta-regression analyses of OS and DFS models are presented. For univariate models, τ²_before and τ²_after denote the between-study variance component before and after inclusion of a single moderator, respectively. CR2-robust p-values (p_CR2) are reported where available. In OS multivariate models, τ²_after ≈ 0 (≈3.44×10⁻¹²) indicates near-complete explanation of between-study heterogeneity by the included moderators. The DFS multivariate comprehensive model failed to converge; CR2-based tests were therefore not performed. An asterisk (*) indicates numerical instability due to an extremely small CR2-robust standard error (SE_CR2 < 1×10⁻¹⁵); the corresponding p-value should be interpreted with caution.

**Abbreviations**: OS, overall survival; DFS, disease-free survival; β, meta-regression coefficient on the log hazard ratio scale; CI, confidence interval; τ², between-study variance component; CR2, cluster-robust variance estimator (type 2); UICC, Union for International Cancer Control; NA, not applicable.
